# Amplicon-based metagenomic survey of microbes associated with the organic and inorganic rhizosphere soil of *Glycine max* L.

**DOI:** 10.1186/s12863-025-01333-2

**Published:** 2025-06-06

**Authors:** Olubukola Oluranti Babalola, Ijeoma Emelda Osuji, Akinlolu Olalekan Akanmu

**Affiliations:** 1 Food Security and Safety Focus Area, Faculty of Natural and Agricultural Sciences, North-West University, Private Bag X2046, Mmabatho, 2735 South Africa; 2 Department of Life Sciences, Imperial College London, Silwood Park Campus, Buckhurst Road, Ascot, Berkshire, SL5 7PY UK

**Keywords:** 16S rRNA gene, Amplicon sequencing, Cow dung, ITS gene, Metagenome, Microbial communities, PCR amplification, Poultry waste, QIIME

## Abstract

**Objectives:**

The metagenomic dataset of 16S rRNA and ITS gene amplicons of DNA were obtained from the cultivated soybean rhizosphere of organic and inorganic treatments. The organic treatments consisted of poultry waste, and cow dung treatments while the inorganic consisted of samples from untreated soybean plots and the bulk. Amplicon sequencing was performed on the Illumina platform, and the raw sequence data were processed and analyzed using Quantitative Insights Into Microbial Ecology (QIIME 2 version 2019.1.).

**Data description:**

The analysis revealed a metagenomic library from soybean rhizospheric soils, providing insights into diversity and distribution of the bacterial and fungal community diversities. The most predominant bacteria phylum taxa across the treatments were Proteobacteria, Firmicutes, Actinobacteriota and Bacteriodota, while those for fungi were Ascomycota, Basidiomycota and Glomeromycota. The dataset provides insights into how different organic fertilization sources affect the structure, composition, and diversity of the microbiome in the soybean rhizosphere. The sequences have been deposited in the Sequence Read Archive (SRA) of the National Center for Biotechnology Information (NCBI) with assigned bioproject accession numbers; 16S rRNA (SRP540791) and ITS (SRP541849).

## Objective

Soybean (*Glycine max* L.) is one of the important legumes across the globe, including South Africa. Its production using organic treatment sources has been encouraged due to their ecofriendly nature [1]. The dataset provides insight into the influence of poultry waste and cow dung as organic soil treatments in soybean cultivation in comparison to the untreated soybean fields. The resulting effects on the community structure and diversity of microbial communities within the soybean rhizosphere of each treatment were evaluated through 16S ribosomal RNA (16S rRNA gene) and Internal transcribe spacer (ITS) amplicon metagenomic sequencing. This dataset explores the diversity, composition, and prevalence of rhizospheric bacteria under different organic and inorganic applications, and the bulk. Raw sequence data available in fastq.gz format have been deposited in the National Center for Biotechnology Information (NCBI) Sequence Read Archive (SRA) database with accession number SRP540791 for 16S rRNA and SRP541849 for ITS sequences. This dataset holds value for identifying novel microbial genes and bioactive compounds that promote plant growth, which could be leveraged to enhance soybean yield, resilience, and sustainable agricultural practices. Additionally, understanding microbial dynamics under different fertilization strategies can aid in developing soil management approaches that support agroecosystem health and productivity [2]. 

## Data description

Rhizospheric soil samples tightly bound to the root surface were collected from the cultivated soybean (*Glycine Max* L.) farm sites at the North-West University farm, Molelwane, Mahikeng (25^0^ 48 11.577’’S,25^0^ 35’ 53.7 62’’E), which has an average rainfall of 540 mm per year. The samples were carried out in the soybean farms under different organic treatments consisting of poultry litter waste, cow dung, untreated soybean plot (control), and the bulk soil. These soil samples were collected in three replicates each, at a 5 cm diameter, and 10-cm depth, and transported in cooler boxes on ice to the laboratory. Samples were weighed while extraction of the whole community Deoxyribonucleic Acid (DNA) was carried out using the NucleoSpin Soil kit (Macherey-Nagel, Germany) by adhering to the manufacture’s instruction. The sequence of the bacterial 16S ribosomal RNA gene was amplified using the universal primer pair regions 16S V4 and 16S V4-V5 with the primers 515F (5'-GTGCCAGCMGCCGCGGTAA-3') and 907R (5'-CCGTCAATTCCTTTGAGTTT-3'), while the internal transcribed spacer regions (ITS1 and ITS2) of the fungal ribosomal DNA were amplified using the primers pair ITS1F (5'-CTTGGTCATTTAGAGGAAGTAA-3') and ITS2 (5'-GCTGCGTTCTTCATCGATGC-3'). Amplicon metagenomic sequencing was carried out using NovaSeq 6000 system (Novogene, Singapore). Quantified libraries were pooled and sequenced on Illumina platforms [3], following standard protocol. Data obtained from this analysis were used to determine the structural diversity and functional profiles of bacteria and fungi in the soybean rhizosphere, across the treatments. The raw sequence dataset in FASTQ format was denoised and chimeras removed using DADA2 plugin within QIIME 2 version 2019.1 [4]. Amplicon sequence variants (ASVs) were classified taxonomically using Silva version 138.1 reference database for 16S rRNA gene [5], while a pre-trained Naïve Bayes classifier using the Unite_INSDC version 8.0 (2018) for ITS [6]. PICRUSt2 [7] and FUNGuild [8] were employed for the functional annotation of the 16S rRNA and ITS genes respectively. Downstream analyses included diversity metrics (alpha and beta diversity), visualization of community composition, and phylogenetic tree construction using the SATé-Enabled Phylogenetic Placement (SEPP) [9] plugin for accurate placement of Amplicon Sequence Variants (ASVs) within the phylogenetic framework. The pipeline facilitated robust and reproducible insights into microbial community structure and function.

The dataset is made up of raw sequence data available in fastq.gz obtained through Amplicon sequencing of soybean rhizosphere metagenome. The sequence Information on dataset and an overview on the microbial community structure are represented in Data file 1. All the obtained datasets in fastq.gz file was deposited at the NCBI-SRA database with accession number SRP540791 and SRP541849 for bacteria (Data set 1) and fungi (Data set 2) respectively in Table 1.

**Table 1:** Overview of data files/data sets

Label	Name of data file/data set	File types (file extension)	Data repository and identifier (DOI or accession number)
*Data file 1*	Sequence information and Microbial community structure	*PDF file (.pdf)*	*Figshare (*10.6084/m9.figshare.28760330*) *[10]
*Data set 1*	Bacterial profilling and functions in soybean rhizosphere under organic fertilization	*fastQ files*	NCBI SRA SRP540791 https://identifiers.org/ncbi/insdc.sra:SRP540791 [11]
*Data set 2*	Fungal profiling and functions in soybean rhizosphere under organic fertilization	*fastQ files*	NCBI SRA SRP541849 https://identifiers.org/ncbi/insdc.sra:SRP541849 [12]

## Limitations

Not applicable.

## Data Availability

The data described in this Data note can be freely and openly accessed on NCBI SRA under https://www.ncbi.nlm.nih.gov/sra/?term=SRP540791 and https://www.ncbi.nlm.nih.gov/sra/?term=SRP541849. Please see Table 1 and references [7 and 8] for details and links to the data.

## Abbreviations

DNA: Deoxyribonucleic Acid
NCBI: National Center for Biotechnology Information
SRA: Sequence Read Archive
DADA2: Divisive Amplicon Denoising Algorithm 2
QIIME: Quantitative Insights into Microbial Ecology
ITS: Internal Transcribed Spacer
16S rRNA gene: 16S ribosomal RNA
SEPP: SATé-enabled Phylogenetic Placement
ASVs: Amplicon Sequence Variants
